# Data for resistance and inductance estimation within a voltage source inverter

**DOI:** 10.1016/j.dib.2019.104104

**Published:** 2019-06-05

**Authors:** Diego Aldana, Yamisleydi Salgueiro, Colin Bellinger, Marco Rivera, César A. Astudillo

**Affiliations:** a Facultad de Ingeniería, Universidad de Talca, Chile; b Data Science for Complex Systems, National Research Council of Canada, Ottawa, Canada

**Keywords:** Voltage source inverter data, Nonintrusive monitoring, High dimensional machine learning

## Abstract

Power converters are essential for the use of renewable energy resources. For example, a photovoltaic system produces DC energy that is transformed into AC by the voltage source inverter (VSI). This power is used by a motor drive that operates at different speeds, generating variable loads. Two parameters, namely, resistance and inductance are essential to correctly adjust the model predictive control (MPC) in a VSI. In this paper, we describe the data from a VSI that incorporates an MPC. We generate four datasets consisting of 399 cases or instances (rows) each one. Two data set comprises the simulations varying the inductance (continuous and discrete versions) and the other two varying the resistance (continuous and discrete versions). The motivation behind this data is to support the design and development of nonintrusive models to predict the resistance and inductance of a VSI under different conditions.

**Table:** Specifications table

Subject area	*Power Electronics*
More specific subject area	*Power Electronic Converters*
Type of data	*Numerical data*
How data was acquired	*The software MATLAB Simulink was used to run simulations of a voltage source inverter with a model predictive control.*
Data format	*Real valued data. 5000 columns plus one for the resistance and another for the inductance. 399 instances in total. CSV format.*
Experimental factors	*Electrical signals of a voltage source inverter with different values of inductance and resistance*
Experimental features	*Simulations of a voltage source inverter with different values of inductance and resistance. Each simulation generated voltage signals as output. Due to the periodicity of the signal, the data collection (inverter output) was shrunk to one-quarter of the signal.*
Data source location	*Chile*
Data accessibility	*Data is available with this article*
Related research article	*Y. Salgueiro-Sicilia* et al. [1]*. Support Vector Machines for Classification of Electrical Resistance Values within a VSI, in 2017 CHILEAN Conference on Electrical, Electronics Engineering, Information and Communication Technologies (CHILECON), Pucon, 2017.* *D. Aldana* et al. [2]*. Performance Assessment of Classification Methods for the Inductance within a VSI, in 2018 IEEE International Conference on Automation/XXIII Congress of the Chilean Association of Automatic Control (ICA-ACCA), Concepcion, Chile, 2018.*

**Table:** 

**Value of the data** • The data can be used to train machine learning models capable of predicting resistance and inductance simultaneously. • The dataset was used as the input for automated learning systems to provide predictions on the behavior of a VSI. • The dataset can be used to verify the reliability of simulations compared to data obtained from real VSI circuits. • As this data possesses a large number of dimensions, it can be used as a good test case for novel dimensionality reduction algorithms.

## 1 Data

**Fig. 1:**
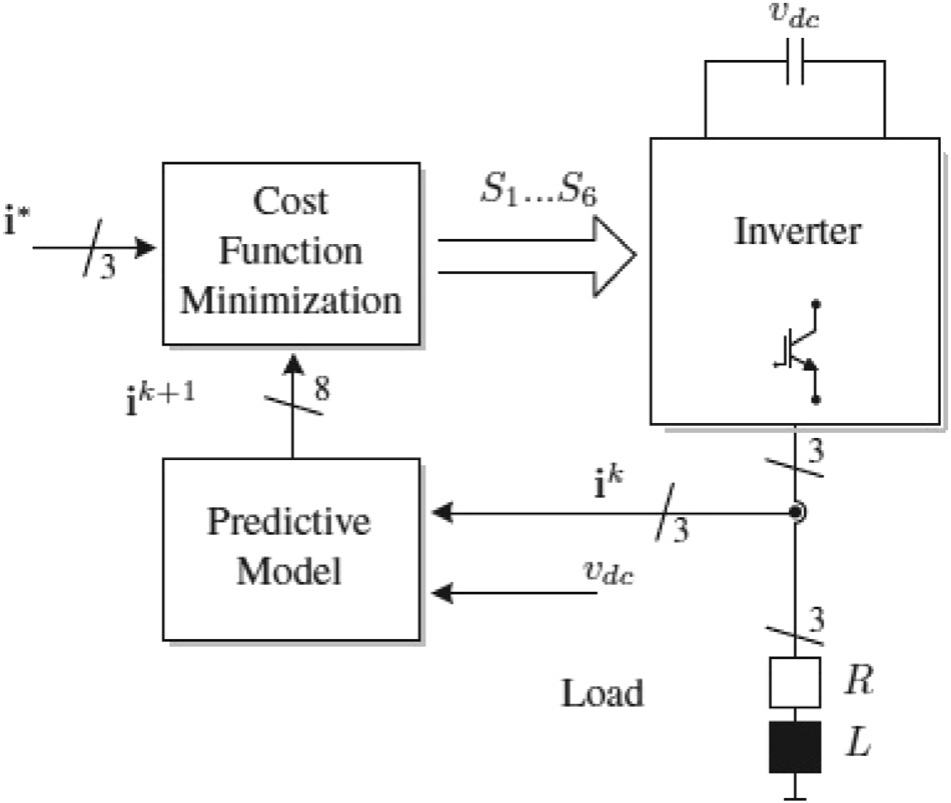
Classic predictive current control method [7].

This dataset contains electrical signals information of a voltage source inverter with a model predictive control (Fig. 1). Two data set comprises the simulations varying the inductance (L) (continuous and discrete versions) and the other two varying the resistance (R) (continuous and discrete versions). The data sets do not present missing or atypical values and the three discrete categories are balanced (i.e., each class has a similar number of instances). The L and R values were simplified performing a discretization by Eqs. (2), (3), respectively. Finally, Fig. 4, Fig. 5, Fig. 6, Fig. 7 present the t-SNE plot for the inductance and resistance in both continuous and discrete values.

## 2 Experimental design, materials, and methods

### 2.1 Voltage source inverter with a model predictive control

Model predictive control (MPC) considers the power converter's finite number of switching states and the mathematical model of the system to predict the behavior of the variables for each switching state. Each prediction is evaluated in with a cost function that selects the switching state that generates the minimum function value [3]. This control strategy has been implemented in different converter topologies and applications such as AC/DC, AC/AC, and DC/AC converters [4], [5], [6].

Fig. 1 shows the general scheme of a two-levels voltage source inverter (2L-VSI) with an MPC where the algorithm steps are [7]:

1. Defining and measuring the values of the current reference $i^{\text{*}}$ and the load current $i^{k}$, respectively.
2. Using the mathematical model of Eq. (1), predict the load current for the next sampling instant $i^{k+1}$ for each valid switching state of the 2L-VSI.

(1) $$i^{k+1}=\left(1-\frac{RT_{s}}{L}\right)i^{k}+\frac{T_{s}}{L}v^{k}$$

3. Considering the cost function ${g\left(k+1\right)=\left(i^{\text{*}}-i^{k+1}\right)}^{2}$, evaluate the error between the load current references and the prediction values for each valid switching state of the 2L-VSI.
4. Select the switching state with the minimum cost function value and apply it to the 2L-VSI in the next sampling time.

Inductance (L) and resistance (R) are relevant parameters to properly set up the MPC cost function. However, their real (true) values vary (atmospheric changes or degradation of the material) causing a deterioration in the effectiveness of the control model.

### 2.2 Data acquisition and preprocessing

**Fig. 2:**
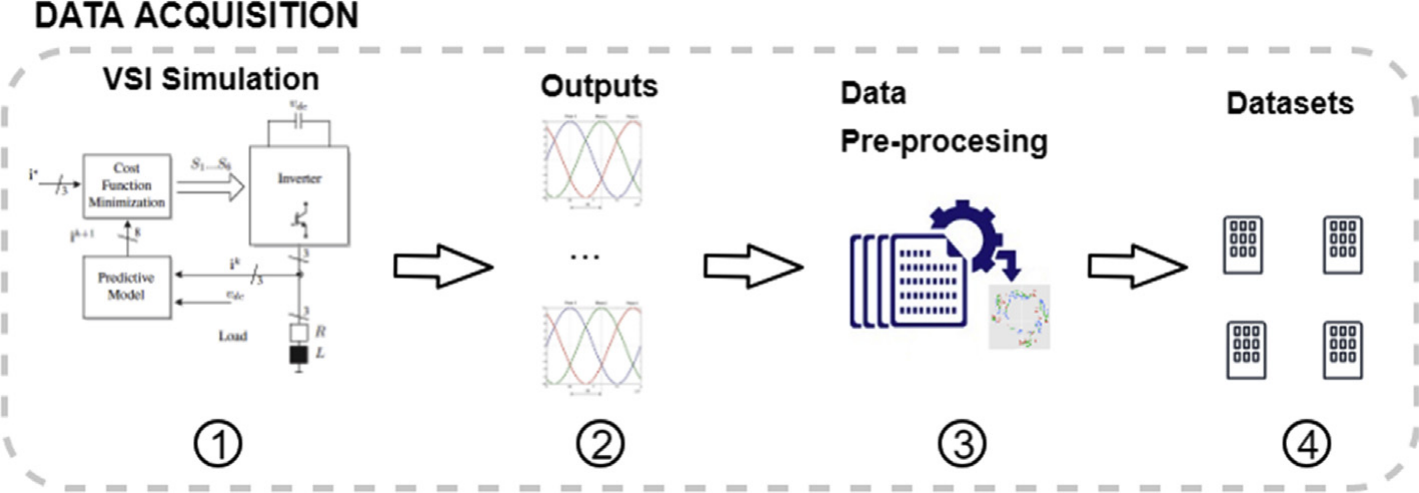
Data acquisition process.

Fig. 2 leads the readers through the steps in the data acquisition process. In step 1, we ran 399 simulations of a voltage source inverter (2L-VSI) with different values of L and R. Each simulation generated current signals as output, Fig. 2 step 2.

**Fig. 3:**
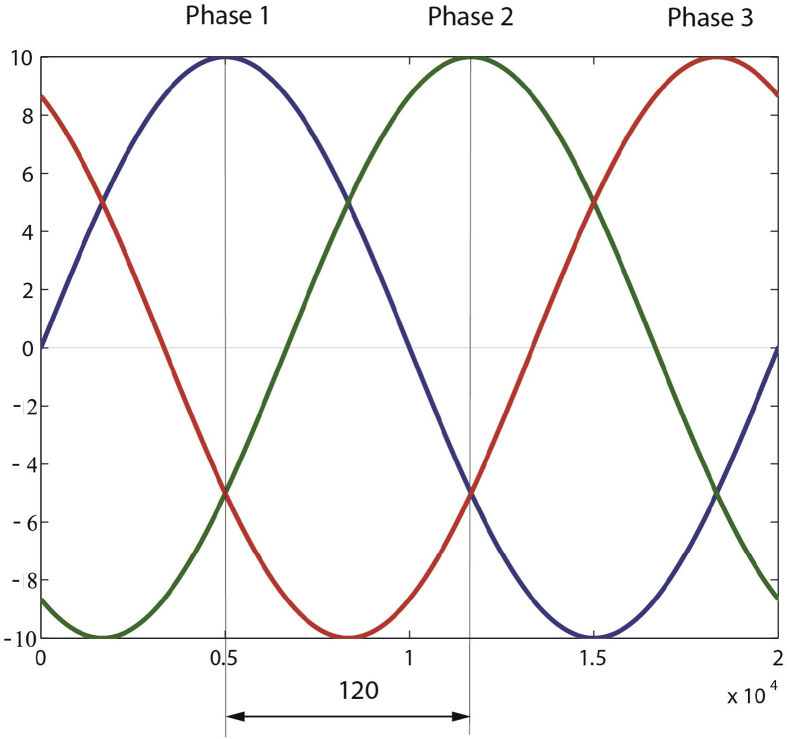
Ideal three phase signal.

Fig. 3 represents an ideal three-phase electrical system, where the phases are equal in frequency and amplitude with a phase difference of 120°. Given the similarity between the phases, it was possible to simplify the problem to the analysis of only one. Additionally, due to the periodicity of the signal, the data collection (converter output) was shrunk to one-quarter of the signal. In other words, to the interval between the origin of the coordinates and the moment in which phase 1 reaches its maximum amplitude.

Therefore, one case contains a total of 5000 attributes (one-quarter of phase 1) and one additional column with the value of the decision variable L or R. Where each attribute corresponds to the current value (Y-axis) at time $T_{i}$ (X-axis).

Further, the L and R values were simplified by performing a discretization according to the following rules (Fig. 2 step 3):

1. We assumed a variation of ±10% from the nominal values of L (301.26 μH) and R (0.30 Ω) and generated the respective intervals.
2. Both intervals were divided evenly into three, substituting the real value of L and R by −1, 0 or 1 if the corresponding value was in the upper, middle or lower third, respectively, Eqs. (2), (3).

(2) $$L\left(x\right)=\;\{\begin{array}{c} -1,\;\;if\;\;\;\;271.1\;\mu H\leq x<291.2\;\mu H\;\;\\ \;\;\;\;\;0,\;\;if\;\;\;\;291.3\;\mu H\leq x<311.3\;\mu H\\ \;\;\;\;1,\;\;if\;\;\;\;311.3\;\mu H\leq x\leq 331.4\;\mu H \end{array}$$

(3) $$R\left(x\right)=\;\{\begin{array}{c} -1,\;\;if\;\;\;\;0.27\;\Omega \leq x<0.29\;\Omega \;\;\\ \;\;\;0,\;\;if\;\;\;\;0.29\;\Omega \leq x<0.31\;\Omega \\ \;\;\;\;\;1,\;\;if\;\;\;\;0.31\;\Omega \leq x\leq 0.33\;\Omega \end{array}$$

With the above simplifications, we generated four datasets consisting of 399 cases or instances (rows) each one, Fig. 2 step 4. Two data set comprises the simulations varying the inductance (continuous and discrete versions) and the other two varying the resistance (continuous and discrete versions). The data sets do not present missing or atypical values and the three discrete categories are balanced (i.e., each class has a similar number of instances).

Data visualization provides a means of gaining a better understanding of the problem and to analyze the behavior of the data. As indicated above, this is a high-dimensional machine learning problem (5000-dimensions). Standard data visualization methods typically only display one to three dimensions. Therefore, a subset of dimensions must be selected, or a low-dimensional representation of high-dimensional datasets must be used. In the present work, this problem was solved by using the t-SNE algorithm [8].

The t-SNE algorithm is a dimensionality reduction technique used to obtain visualizations of data with high dimensionality. The method works by mapping the different high dimension instances into new instances with low dimension while keeping the similarities found in the original data.

**Fig. 4:**
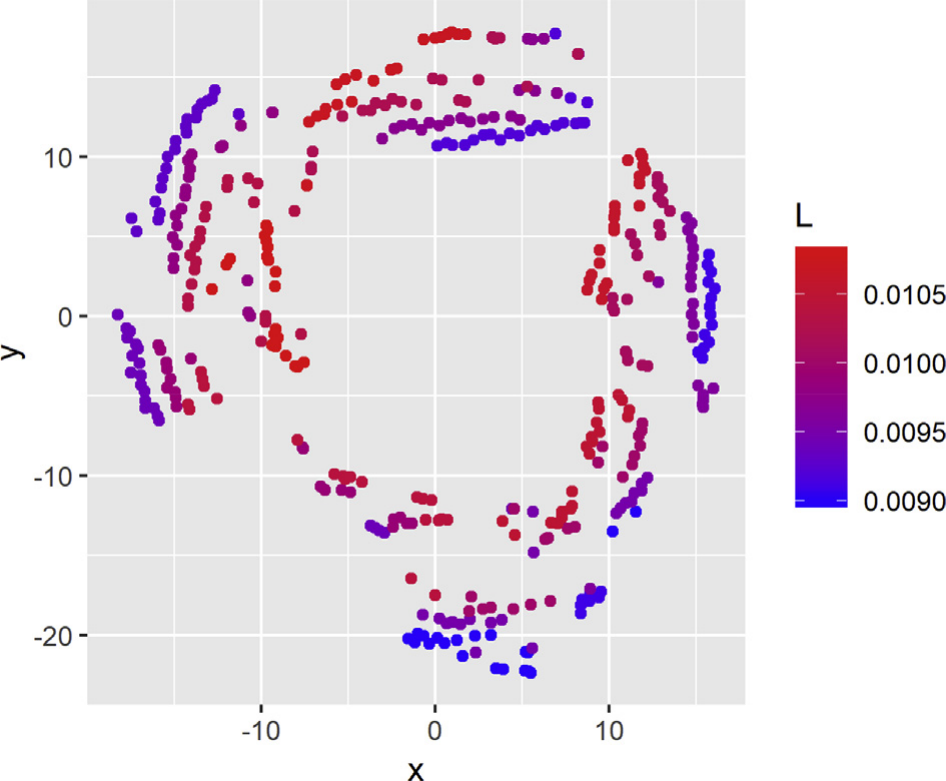
t-SNE visualization for inductance.

**Fig. 5:**
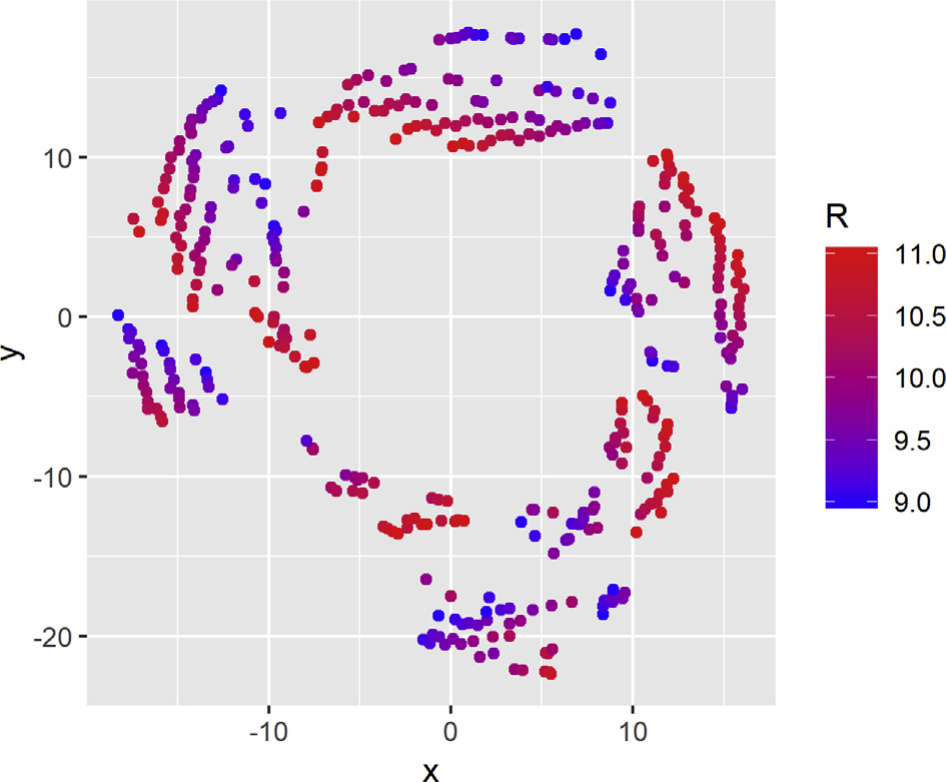
t-SNE visualization for resistance.

The t-SNE plot for the inductance variable is depicted in Fig. 4. Each point represents a different simulation and concentrates 5000 current while the color of the point represents the inductance value. Bluish colors identify lower inductance values, and red is used to indicate higher inductance values. Additionally, Fig. 5 shows a similar plot but detailing the resistance values.

**Fig. 6:**
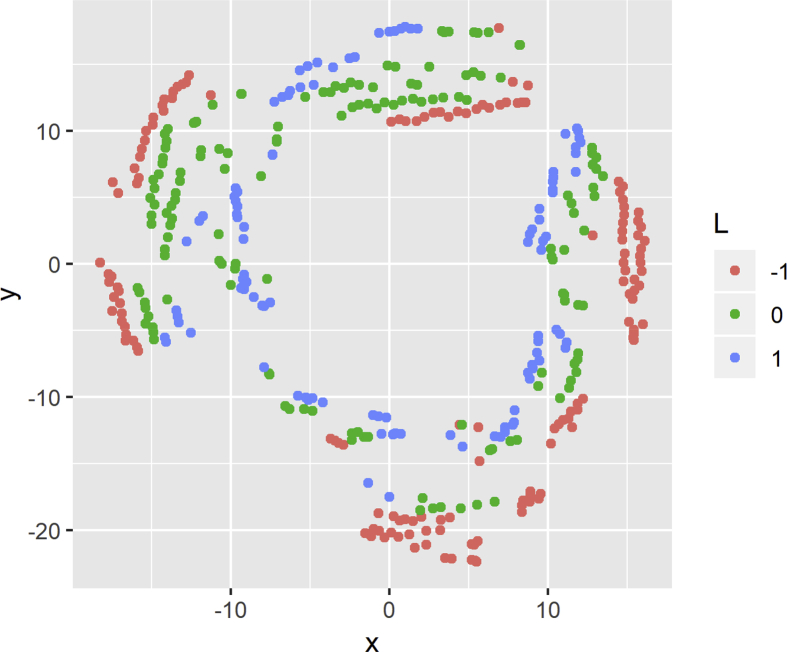
t-SNE visualization for discretized inductance.

**Fig. 7:**
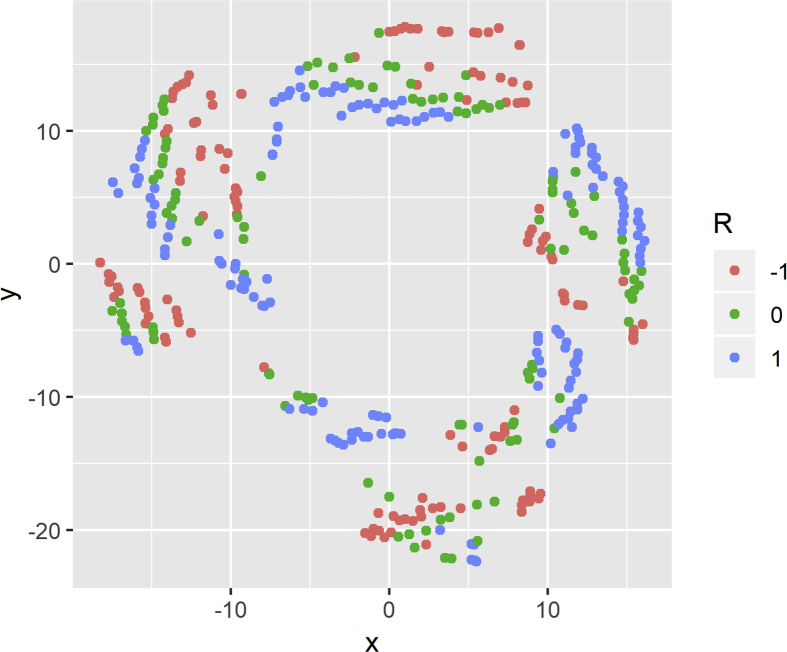
t-SNE visualization for discretized resistance.

Fig. 6 shows the visualizations obtained with t-SNE colored according to the discrete value of the inductance. The points belong to three different colors (green, red and blue) depending on the discrete value obtained using Eq. (1), where red represents a discrete value of −1, green represents a value of 0, and blue is a value of 1. Analogously, Fig. 7 shows the t-SNE visualization for the resistance. The colors are assigned according to the values obtained by Eq. (2).
